# Native and Recombinant Yeast Producers of Lactic Acid: Characteristics and Perspectives

**DOI:** 10.3390/ijms26052007

**Published:** 2025-02-25

**Authors:** Aksyniia Tsaruk, Kamila Filip, Andriy Sibirny, Justyna Ruchala

**Affiliations:** 1 Faculty of Biotechnology, Collegium Medicum, University of Rzeszow, 35-601 Rzeszow, Poland; 2 The Doctoral School of the University of Rzeszow, University of Rzeszow, 35-959 Rzeszow, Poland; 3 Department of Molecular Genetics and Biotechnology, Institute of Cell Biology NAS of Ukraine, 79005 Lviv, Ukraine

**Keywords:** lactic acid, polylactic acid, renewable resources, metabolic engineering, yeasts

## Abstract

Lactic acid (LA) is a key chemical used in various industries, including food, pharmaceuticals, and bioplastics. Although traditionally produced using lactic acid bacteria, yeasts offer significant advantages, such as higher tolerance to acidic environments, a broader substrate range, and the potential for genetic and metabolic engineering. This review explores the potential use of *Lachancea thermotolerans*, *Saccharomyces cerevisiae*, *Kluyveromyces marxianus*, *Kluyveromyces lactis*, *Candida utilis*, and *Pichia kudriavzevii* as LA producers, highlighting their unique characteristics and industrial applications. *S. cerevisiae* stands out due to its robust genetic toolkit and acid tolerance, while *K. marxianus* offers thermotolerance and the efficient utilization of lactose and pentoses, making it ideal for high-temperature fermentations. *K. lactis* is particularly suited for valorizing dairy by-products like whey, *P. kudriavzevii* exhibits high tolerance to multiple stresses, while *C. utilis* demonstrates superior resilience to lignocellulosic inhibitors, enabling its use in biorefineries. Key challenges, including enhancing LA tolerance and optimizing metabolic pathways, are addressed through strategies like heterologous lactate dehydrogenase (LDH) expression, redox balance modification, and adaptive laboratory evolution. The review also discusses industrial applications, particularly in the context of circular economy approaches, where yeasts can convert waste streams into high-value LA. Future research should focus on integrating yeasts into scalable, sustainable bioprocesses to meet the growing demand for renewable and biodegradable materials.

## 1. Introduction

Lactic acid (LA) is a 2-hydroxycarboxylic acid widely occurring in fermented fruits, vegetables, milk, and animal muscle tissue in two enantiomeric forms (L- and D-lactic acid) as a product of anaerobic metabolism [1]. It was discovered for the first time in 1782 by the Swedish scientist Karl Wilhelm Sheele in sour milk [2]. Microorganisms produce both L and D-enantiomers (L-LA and D-LA respectively), whereas mammals produce only L-isomer. LA has several properties that make it a useful compound for multiple products in the textile, chemical, cosmetic, pharmaceutical, and food industries. Depending on the application, LA is needed in one of the two forms, or as a racemic mixture [1,3].

As LA (more precisely, L-LA) is non-toxic, it has been recognized by the United States Food and Drug Administration as GRAS (Generally Recognized as Safe). LA can be found in dairy products, meat, and alcoholic beverages like wine or beer as a preservative, flavor-enhancer, and acidity regulator. LA is added to cosmetics due to its moisturizing, antimicrobial, and exfoliating effects on the skin [4].

High demand for LA production lies not only in an expanding market of fermented food, or skincare and hair products, but also in a growing need for biodegradable and renewable polymer production. LA is a component of synthetic aliphatic polyester called polylactic acid (PLA), which is a primary driver of the global LA market nowadays. PLA can be synthesized by the direct condensation polymerization of LA produced via fermentation or through ring-opening polymerization (ROP) [5,6]. PLA polymers are biodegradable, which makes them a good alternative to synthetic plastic for use in packaging, utensils, textiles, and medical supplies production. Furthermore, due to a lack of toxicity and biocompatibility, PLA has also found applications in drug delivery, tissue engineering, and implants [7,8]. The advantages of PLA over traditional plastic entail the use of renewable sources for its production and lower energy consumption by 65%. Although PLA exhibits greater mechanical strength compared to other biopolymers, it has notable limitations, including reduced durability and flexibility compared to petrochemical plastics. Additionally, PLA demonstrates significant shrinkage under heat, which restricts its use in applications requiring thermal stability [9]. Nevertheless, the expansion of PLA applications, particularly in response to increasing regulatory and consumer demand for sustainable materials, underpins the growth of the LA market.

The application of PLA-based polymers aligns with global trends emphasizing the replacement of petrochemical plastics with renewable, sustainable materials, as supported by policies like the European Union’s regulations on reducing plastic waste [10,11]. Reaching roughly 140,000 tons per year, PLA is the most widely used biopolymer in the food packaging industry [9]. The PLA market is constantly growing due to increasing consumer awareness and new policies, like the European Union’s regulations demanding the substitution of traditional plastics with more ecological and biodegradable materials [12]. According to a market analysis report by Grand View Research, in 2023, the global LA market reached USD 3.37 billion, and it is predicted to grow by approximately 8% annually by 2030 [13]. Other source data indicate that in 2015, global LA production was 700 kilotons, and in 2022 the number increased to 1500 kilotons, and the forecasts predict an increase to approximately 2800 kilotons in 2030 [14].

The chemical synthesis of LA includes processes such as the hydrolysis of lactonitrile, the base-catalyzed degradation of sugars, or the nitric acid oxidation of propylene. However, those methods can only give a racemic mixture of LA, and are not efficient or economically profitable [15,16].

**Figure 1:**
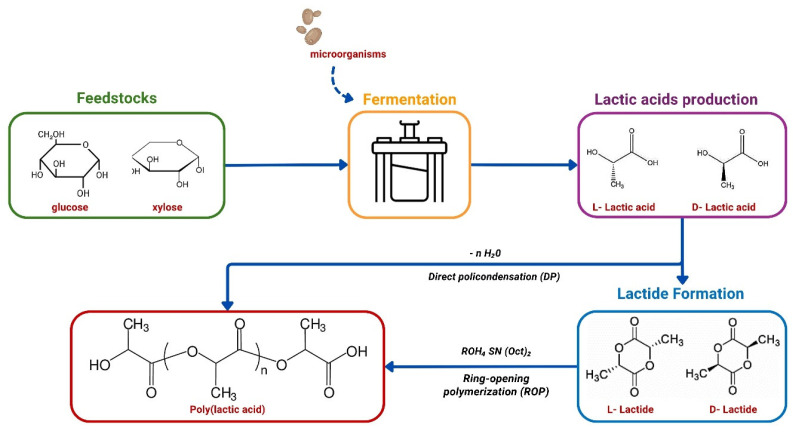
Process flow for lactic acid production and polymerization (own elaboration).

LA is produced by lactic acid bacteria (LAB) from the genera *Lactobacillus*, *Lactococcus*, *Streptococcus*, and *Bacillus*, and filamentous fungi like *Rhizopus nigricans*, *Rhizopus oryzae* and *Mucor* [17,18,19,20,21]. Another microorganism capable of LA fermentation is the yeast *Lachancea thermotolerans* [22,23]. The carbohydrates preferred for LA production should ideally consist of hexoses or pentoses (Figure 1). Importantly, for microbial LA fermentation on a large scale, the use of pure sugars as substrates is not economical. The production efficacy may be improved by the utilization of raw materials like molasses, beet juice, corn syrup, date juice, whey, or lignocellulose.

Fungi and bacteria are the most widely used microorganisms for LA production via fermentation. Approximately 90% of commercially produced LA is synthesized by LAB. These bacteria contain two key enzymes—L-lactate dehydrogenase (L-LDH) and D-lactate-dehydrogenase (D-LDH). LA of bacterial origin is usually a racemic mixture [4]. Yet, for the polymerization process, the industry demands a product consisting of only one enantiomer. Similarly, due to the toxicity of D-LA, only pure L-LA is suitable for food and pharmaceutical applications [24]. There are already genetically modified bacteria that have been created and are capable of producing high amounts of one of the enantiomers. In those LAB, either *L-LDH* overexpression or *D-LDH* deletion have been introduced [1,4]. In some matters, fungi seem to be a better choice for industrial LA production. For example, *Rhizopus* grows on synthetic media like an inorganic nitrogen source, a less complicated medium than the one LAB need. *Rhizopus* synthesizes only L-LA, which is preferred for polymer production; the mycelium formation makes biomass separation easier, and thanks to the extracellular amylases, starchy materials may be hydrolyzed without previous preparation. On the other hand, LAB produce higher yields of LA than fungi. LA inhibits the growth of most bacteria, whereas *Rhizopus* tolerates the high acidity of the environment, which lowers the risk of contamination [20,25].

In our review, we focused on the production of LA by yeast because it presents a compelling alternative to LAB for industrial LA production, especially in scenarios demanding robustness, substrate flexibility, and high efficiency. While LAB remain dominant in traditional food fermentations, the superior tolerance, scalability, and engineering potential of yeasts make them increasingly attractive for modern industrial applications. Future advancements in metabolic engineering and bioprocess optimization will likely further cement the role of yeasts as primary microbial factories for LA and related products.

## 2. *Lachancea thermotolerans*—Natural Yeast Producer of LA

*L. thermotolerans* is a unique natural LA producer among yeasts, and may have the potential to be an excellent source of pure L-LA in industry. This microorganism has the unique ability to ferment sugars into both ethanol and L-LA. In winemaking, this feature leads to an increase in acidity, and has a positive influence on the aroma, color, and flavor of a wine [26].

*L. thermotolerans* has three isoenzymes of lactate dehydrogenase (LDH)—Ldh1, Ldh2, and Ldh3. LDH belongs to the L-lactate/L-malate dehydrogenase super-family. The reduction of pyruvate to L-LA by LDH is another way to regenerate NAD^+^, thus limiting the conversion of the pyruvate to ethanol. Two genes encoding the LDH enzyme, *LDH2* and *LDH3*, are in tandem, located on the same chromosome G, whereas *LDH1* is located on chromosome D. Of all three isoenzymes, LDH2 is more likely to have the greatest impact on L-LA production. It has been found that the transcription profiles of ldh1 and ldh3 are very similar among different strains differing in L-LA production. Only the *ldh2* expression level is correlated to L-LA accumulation levels because increased transcription levels of *ldh2* were documented in strains with the highest L-LA yields. Single nucleotide mutations in promotor sequences of *ldh* genes, especially in the TATA box area, lead to changes in regulatory machinery. This may lead to the higher transcription of the LDH [22].

*L. thermotolerans* belongs to the group of yeasts that exhibit the Crabtree effect. Those kinds of yeasts, in aerobic conditions with high glucose concentrations, prefer generating energy via the fermentation process rather than via complete glucose oxidation, which is a more efficient mode of generating ATP. Moreover, those yeasts in such an environment tend to produce more ethanol than biomass [27,28]. Shekhawat et al. described the response of *L. thermotolerans* yeast to anaerobic conditions. The transcriptomic analysis showed the upregulation of genes involved in the glycolysis and fermentation process with simultaneous downregulation of the tricarboxylic cycle and pentose phosphate pathway. Furthermore, all three *ldh* homologs were upregulated, while only two alcohol dehydrogenases were activated, whereas in S. cerevisiae, four were active in comparable conditions. This study has given evidence of the switch to higher L-LA production by *L. thermotolerans* under oxygen deprivation [29]. The LA production may be directly influenced by the presence of nitrogen in media. Battjes et al. reported on the impact of nitrogen concentration on LA production. They also observed higher LA fermentation in anaerobic conditions and a lower biomass rate correlated with high LA production [30]. No attempts to engineer *L. thermotolerans* strains with enhanced LA production have been reported so far.

Another way to increase LA yield is the addition of CaCO_3_ to fermentative media. Buffering media with the CaCO_3_ leads to pH stabilization and increased LA production. Studies on *Lactobacillus* species have shown an augmentation of LA by up to 17% compared with the cultures without CaCO_3_ [31,32,33]. However, the presence of CaCO_3_ slows down LA productivity, which is probably a result of the negative impact of Ca^2+^, and additionally, high concentrations of CaCO_3_ cause a drop in LA production [31,34]. LDH, required for pyruvate conversion to LA, tends to work slower in more acidic environments than pyruvate decarboxylase. In this scenario, ethanol production is higher than LA due to the advantage of pyruvate conversion to ethanol rather than LA. The addition of CaCO_3_ to fermentation media increases LA production but also leads to calcium lactate [Ca(LA)_2_] formation, which means that LA has to be recovered from the media. Similar studies conducted on *R. oryzae* have shown that the addition of CaCO_3_ increases LA yields by up to 50% [35,36].

## 3. The Most Popular Genetically Modified Yeasts for LA Production

### 3.1. Saccharomyces cerevisiae

*S. cerevisiae* has emerged as a promising host for LA production due to its GRAS status, well-characterized genetics, and ability to thrive in acidic environments (pH < 4). These traits make it suitable for large-scale industrial processes, particularly where contamination risks or low-pH conditions challenge other microorganisms. *S. cerevisiae* has been extensively studied and offers a wide range of genetic tools for metabolic engineering [37,38]. Thanks to the easiness of performing genetic manipulations, *S. cerevisiae* is a great target for different biotechnological purposes [39,40].

*S. cerevisiae* prefers glucose over other sugar sources, and despite the presence of oxygen, it favors fermentative ethanol and other two-carbon compound production over the oxidative respiration pathway, even though it is not the most effective way to produce ATP (Crabtree effect) [39]. Interestingly, the produced ethanol may become a substrate. This metabolism shift (“diauxic shift”) happens when glucose depletes, and oxygen is present, which allows the regaining of energy “wasted” on fermentation, known as the “make–accumulate–consume” strategy. Furthermore, ethanol production, which is toxic to most microorganisms, enables the efficient elimination of microbial contamination [41].

Another advantage of *S. cerevisiae* is the ability of the engineered strains to grow on alternative substrates, including industrial by-products like glycerol. This versatility aligns with sustainable production goals, enabling the efficient utilization of renewable feedstocks (see Section 6). The main challenge in utilizing *S. cerevisiae* for LA production is its natural preference for ethanol fermentation. Overcoming this limitation requires metabolic engineering strategies, including the introduction of heterologous *LDH* genes and the disruption of ethanol biosynthesis pathways. These strategies, alongside redox optimization, are discussed in detail in Section 5.1. Despite their advantages, *S. cerevisiae* is still unable to achieve a high LA titer or does not exhibit resistance to its high concentrations, making further genetic modifications necessary to improve these parameters. Approaches like adaptive laboratory evolution have shown promise in addressing these limitations, and are described in Section 6. With ongoing advancements, *S. cerevisiae* continues to be a leading candidate for LA production, particularly in industries seeking robust, genetically tractable organisms.

### 3.2. Komagataella phaffii

*Komagataella phaffii* (formerly *Pichia pastoris*) belongs to the *Saccharomycetales* and possesses GRAS status. It can grow on various carbon sources like methanol, glucose, trehalose, L-rhamnose, ethanol, mannitol, glycerol, sorbitol, LA, and acetic acid. Methylotrophy relies on an assimilation pathway localized in largely expanded peroxisomes, and a cytosolic methanol dissimilation pathway [42,43]. Thanks to its haploid nature, ease of genetic manipulation, efficient synthesis of heterologous proteins, and ability to use diverse carbon sources, *K. phaffii* is a versatile system that can be used for industrial applications, including bio-manufacturing and high-value chemical production [44].

Due to its natural capacity to grow on glycerol and methanol, *K. phaffii* might be used for the utilization of industrial waste, such as crude glycerol, the main by-product of biodiesel production. For every 10 kg of biodiesel, approximately 1 kg of glycerol is produced [45]. Considering the global market of biodiesel production, which in 2022 reached 52 million tons [46], the use of glycerol as a substrate for LA production by *K. phaffii* adds to the biodiesel production chain. *K. phaffii* with an incorporated heterologous *LDH* gene can produce up to 24 g/L of L-LA from glycerol [47,48,49]. Similarly, D-LA production was achieved using methanol as a carbon source [50]. The titer of obtained D-LA ranged up to 17 g/L. However, challenges remain in optimizing LA production, particularly in balancing growth and product formation under aerobic conditions. Future research should focus on integrating *K. phaffii* into biorefinery models, where it can co-produce LA alongside other valuable compounds, such as biosurfactants or enzymes. Advances in process engineering, including the real-time control of methanol concentrations and enhanced tolerance to LA, will further enhance its industrial relevance.

### 3.3. Kluyveromyces marxianus

*Kluyveromyces marxianus* is a nonconventional yeast belonging to the family *Saccharomycetaceae*. *K. marxianus* grows at temperatures up to 52 °C, making it ideal for high-temperature fermentation processes with reduced contamination risks. *K. marxianus* stands out as a promising organism for LA production due to its thermotolerance and ability to metabolize a wide range of substrates, including lactose. These traits make it particularly suitable for the dairy industry, where it can valorize whey, a by-product of cheese production, into valuable chemicals like LA [51,52,53]. Thus, engineering *K. marxianus* for LA production allows a reduction of cost in the manufacturing process [54,55,56]. Future research should explore its ability to co-ferment diverse substrates and its application in integrated biorefinery models. With ongoing advancements, *K. marxianus* may become a key player in sustainable LA production, particularly in industries prioritizing waste valorization. Recent studies have shown that genetically modified strains of *K. marxianus* can efficiently convert sugars from hydrolysates into LA while tolerating inhibitors typically present in these substrates, such as furfural and hydroxymethylfurfural (HMF). This capability positions *K. marxianus* as a viable candidate for sustainable LA production in biorefineries utilizing agricultural residues or forestry waste.

### 3.4. Kluyveromyces lactis

*Kluyveromyces lactis* was for many years not considered a candidate for use in the biotechnological industry. Only in the 1980s did *K. lactis* start gaining popularity as a recombinant protein producer. Among the two varieties of *K. lactis*, the domestic and the wild, the domestic variety *K. lactis var. lactis* is mainly employed in the biotechnological industry [57]. *K. lactis* can utilize glucose, xylose, xylitol, cellobiose, arabinose, and lactose. *K. lactis* is an important yeast species in the context of LA production, particularly due to its ability to efficiently metabolize lactose. This characteristic makes it a valuable candidate for the dairy industry, where lactose-rich waste products such as whey are abundant. Compared to its close relative *K. marxianus*, *K. lactis* operates optimally at moderate temperatures (~30 °C), providing energy savings in fermentation processes and making it suitable for applications where the precise control of environmental conditions is required [23,58]. However, *K. lactis* faces specific challenges in LA production. Its natural tolerance to LA is limited compared to species like *S. cerevisiae* or *K. marxianus*, necessitating metabolic engineering to enhance acid resistance and productivity. Genetic modifications have focused on introducing heterologous LDH genes and optimizing carbon flux toward LA. For instance, engineered strains of *K. lactis* have demonstrated high L-LA yields (Table 1), showcasing its potential as an industrial LA producer [59]. Moreover, just like other mentioned yeasts, *K. lactis* can be cultivated on inexpensive media, which decreases the costs of industrial processes [59].

### 3.5. Candida utilis

*Candida utilis* is a versatile yeast species with the unique ability to utilize a wide range of carbon sources, including pentoses like xylose and unconventional substrates such as ethanol and glycerol. This makes it particularly suitable for use by biorefineries aiming to valorize lignocellulosic biomass or industrial waste streams. Unlike *S. cerevisiae*, *C. utilis* demonstrates higher tolerance to inhibitors like furfural and hydroxymethylfurfural (HMF), which are common in hydrolysates of lignocellulose [82]. In the context of LA production, *C. utilis* offers potential advantages in processes where mixed or low-quality substrates are used. For example, its ability to grow on nutrient-poor media reduces the overall costs of fermentation. Engineered strains of *C. utilis* have demonstrated promising LA yields, particularly when utilizing lignocellulosic hydrolysates, highlighting its role in sustainable production systems. Moreover, due to efficient fermentation and genetic engineering, *C. utilis* can produce great amounts of metabolites like ethanol or isopropanol [82]. Another *C. utilis* feature that makes it great for biotechnological and industrial usage is the ability to use nitrate as a nitrogen source, which makes *C. utilis* more resistant to poor conditions than other yeast [83]. However, challenges remain in optimizing *C. utilis* for industrial LA production. Compared to *S. cerevisiae*, it has less developed genetic tools, making metabolic engineering more complicated. Recent advances have focused on introducing heterologous LDH genes and enhancing redox balance to increase LA productivity. Additionally, optimizing fermentation conditions—such as maintaining efficient aerobic growth while promoting LA synthesis—will be crucial for its industrial scalability. Tolerance to low pH, robust fermentation, effective genetic modifications, low-cost media, and a large spectrum of utilized substances make a metabolically engineered *C. utilis* an excellent candidate for LA production on xylose; however, glucose conversion into LA is more efficient [62].

## 4. Lactic Acid Metabolism

**Figure 2:**
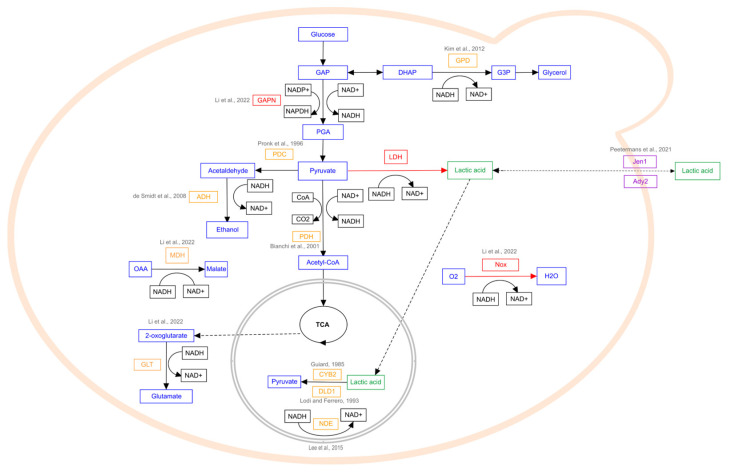
Simplified pathways of LA metabolism in engineered yeast strains. Endogenous metabolic pathways are black (solid line), transport pathways are black (dashed line), endogenous enzymes are orange, heterogeneous genes are red, LA is green, and transporters are purple. LDH, lactate dehydrogenase; PDC, pyruvate decarboxylase; ADH, ethanol dehydrogenase; GAP, glyceraldehyde 3-phosphate; DHAP, dihydroxyacetone phosphate; PGA, 3-phosphoglyceric acid; GPD, glycerol phosphate dehydrogenase; PDH, pyruvate dehydrogenase; NAD^+^/NADH, nicotinamide adenine dinucleotide; NADP^+^/NADPH, nicotinamide adenine dinucleotide phosphate; GAPN, NADP^+^-dependent 3-phosphate glyceraldehyde dehydrogenase; Nox, NADH oxidase; GLT, glutamate synthase; MDH, malate dehydrogenase; Jen1 and Ady2, monocarboxylate transmembrane transporters; CYB2, cytochrome b2; DLD1, D-lactate dehydrogenase; NDE, NADH dehydrogenase; OAA, oxaloacetate [59,76,78,85,86,87,88,89,90,91].

In yeasts, hexoses (mainly glucose) are metabolized through the glycolysis pathway to pyruvate (Figure 2). The presence of endogenous LDH in *L. thermotolerans* allows the reduction of pyruvate directly to L-LA, along with NAD⁺ regeneration from NADH. Other main products of fermentative metabolism in *L. thermotolerans* include ethanol and acetate [84]. Further, LA could be transported outside of the cell by Jen1 and Ady2 monocarboxylate transmembrane transporters, or converted back to pyruvate by the cytochrome b2 (Cyb2) and D-lactate dehydrogenase Dld1 located in mitochondria [76,77,85].

In other yeast species, due to the lack of LDH, the main product of fermentative metabolism is ethanol, which is synthesized from pyruvate through a two-step enzymatic process catalyzed by pyruvate decarboxylase (PDC) and alcohol dehydrogenase (ADH). Thus, in most cases, LA production requires not only the introduction of LDH, but also the disruption of a range of pathways leading to the formation of various byproducts, such as glycerol, which is produced from a glycolysis intermediate; glyceraldehyde-3-phosphate (GAP), due to the activity of glycerol phosphate dehydrogenase (GPD); and acetyl-CoA, produced from pyruvate in a reaction catalyzed by pyruvate dehydrogenase (PDH) [59,86]. The indicated reactions involve the reduction and regeneration of NAD^+^; alterations of these reactions demand modifications of other pathways to maintain cellular redox balance. This can be achieved by the overexpression or deletion of enzymes involved in other redox reactions. For example, NAD^+^ regeneration occurs in reactions catalyzed by endogenous glutamate synthase (GLT), malate dehydrogenase (MDH), NADH dehydrogenase (NDE), or heterologously expressed NADH oxidase. Another approach includes creating a cofactor competing system through an expression of heterologous NADPH-dependent 3-phosphate glyceraldehyde dehydrogenase, which converts glycolysis metabolite glyceraldehyde-3-phosphate (GAP) to 3-phosphoglyceric acid (PGA), while the native enzyme involved in this reaction is NAD^+^-dependent [76,78].

## 5. Primary Targets for Metabolic Engineering of LA Producers in Yeast

Since LA biosynthesis is extremely rare among yeasts and the amounts of produced lactate are low compared to those in LAB, metabolic engineering approaches are required to obtain efficient LA-producing yeast strains (Table 1). This objective can be accomplished by introducing heterologous *LDH* genes for redirecting ethanol biosynthesis toward LA production. Additionally, other modifications might be introduced into engineered yeast strains to maximize LA yield and production. This includes disrupting metabolic pathways of byproducts and competing compounds, altering transport pathways, and improving acid tolerance through adaptive laboratory evolution (Figure 2).

Among the main traits that make yeasts attractive organisms for LA production is tolerance to environmental factors such as high temperatures, low pH, and phage infections. The resistance to acidic environments precludes the need to use large amounts of neutralizing agents to buffer the medium during fermentation. The ability to grow and ferment substrates at high temperatures reduces the need for intensive cooling of the medium, and lowers the risk of contamination in industrial conditions. Moreover, yeasts do not require complex or expensive growth media and are able to ferment a variety of substrates. This review specifically focuses on metabolic engineering targets tailored for enhancing LA production in yeast, with a comprehensive overview of strategies reported in research over the last 30 years. Unlike broader reviews that cover LA production across various microorganisms [4,16], this article provides targeted insights into yeast as a production host, highlighting unique challenges and advancements that may not apply to other organisms.

The first attempt at engineering LA-producing yeast strains was carried out in 1994 [71]. The obtained strain of *S. cerevisiae* expressing the bacterial L-LDH gene was able to achieve mixed fermentation, producing both 17 g/L of ethanol and 12 g/L of L-LA. Such strains have potential applications in the processes that require biological acidification in alcoholic fermentation. However, obtaining yeast strains that do not produce, or produce minimal amounts of, by-products and achieving homolactic fermentation are the goals of engineering efficient LA producers. These can be achieved by the outright elimination or partial inhibition of competing metabolic pathways. The most frequently reported modifications and their effects are described below.

### 5.1. Number of LDH Copies

Numerous studies have been conducted to explore the effects of increasing the number of copies of the *LDH* genes. In *S. cerevisiae*, the introduction of six copies of the *LDH* gene resulted in L-LA production at 68 g/L, which is 1.28 times greater than the amount of L-LA produced by the strain with only two copies of the corresponding gene [92]. The LDH activity also increased by 2.8 times in the strain with six *LDH* copies.

A similar impact of a higher *LDH* copy number on L-LA production was demonstrated on engineered *C. utilis* strains. The strain harboring two copies of the corresponding gene had a higher L-LA production rate compared to the strain with only one copy [62].

In *Candida sonorensis*, increasing the number of *LDH* copies from one to three has led to higher LDH activity, increased L-LA yield, and reduced ethanol production, although volumetric L-LA production remained unchanged [61].

The engineered *K. marxianus* strain simultaneously expressing *LDH* genes from *Staphylococcus epidermidis*, and *Lactobacillus acidophilus* demonstrated an L-LA production of 16 g/L, while on the contrary, the expression of either one of those genes resulted in L-LA productions of 8.4 g/L and 6.8 g/L, respectively [56]. This finding implies that LA production in yeast could be elevated by the heterologous co-expression of *LDH* genes characterized by different optimal pH values. The mentioned approach aims to supply LDH activity during fermentation when pH values are oscillating.

### 5.2. Altering Ethanol Biosynthesis Pathways

#### 5.2.1. Pyruvate Decarboxylase

One of the prevailing approaches is to disrupt the genes coding for key enzymes involved in ethanol biosynthesis. One of those crucial enzymes in ethanol fermentation is pyruvate decarboxylase (PDC; EC 4.1.1.1), which converts pyruvate to acetaldehyde. In *S. cerevisiae*, six *PDC* genes have been described: structural genes *PDC1*, *PDC5*, and *PDC6* coding for catalytic proteins, and functional genes *PDC2*, *PDC3*, and *PDC4* coding for regulatory proteins [88]. *PDC1* accounts for most of the PDC activity, while *PDC5* demonstrates activity only in *∆pdc1* mutants [93]. On the contrary, the deletion of *PDC6* does not cause significant changes in PDC activity [94].

The deletion of *PDC* genes redirects pyruvate conversion to LA instead of ethanol. The single deletion of the *PDC1* gene or the double deletion of the *PDC1* and *PDC5* genes in L-LA-producing *S. cerevisiae* somewhat increases L-LA production; however, ethanol is still produced in sufficient amounts [74,95]. Meanwhile, L-LA production in engineered *S. cerevisiae* with the triple deletion of the *PDC1*, *PDC2*, and *PDC5* is lower compared to the strain with active *PDC* [96]. *S. cerevisiae* with deleted *PDC1* and *PDC5* genes, or deleted *PDC1*, *PDC5*, and *PDC6* genes, demonstrated an inability to ferment glucose, and consequently impaired growth on glucose medium [97]. This observation can be explained by the fact that acetaldehyde produced by Pdc enzymes serves as a precursor for acetyl-CoA, which is essential for fatty acid production [97]. Therefore, current metabolic engineering approaches aim to decrease PDC activity rather than eliminate it.

The first example of the complete replacement of ethanol fermentation with L-LA fermentation in yeast was the engineered *K. lactis* strain, with the heterologous expression of the bovine *LDH* gene and the deletion of *PDC* gene [66]. In contrast to *S. cerevisiae*, *K. lactis* has one *PDC* gene (*KlPDCA*), the deletion of which does not result in impaired growth on glucose; however, *∆pdcA* mutants do not demonstrate PDC activity and ethanol production [98]. The amount of produced L-LA reached 109 g/L in bioreactor fermentation on a glucose-based medium. The same strategy was applied to other engineered LA-producing yeast strains (Table 1) [76,77].

Of two genes coding for PDC in *C. sonorensis*, *PDC2* is responsible for most PDC activity. Its disruption inhibits growth rate and PDC activity, while the deletion of *PDC1* does not have the same effect. However, the complete elimination of PDC activity requires the knockout of both genes. The heterologous expression of bacterial *LDH* in the *C. sonorensis* strain with abolished ethanol biosynthesis due to the deletion of both *PDC* genes resulted in L-LA production up to 92 g/L on YNB + 10% glucose buffered with CaCO_3_ [61].

An L-LA titer of 86 g/L at a yield of 1 g/g glucose was achieved in *Candida boidinii*-expressing bovine *LDH* under the control of native *PDC1* promoter in a *PDC1* disrupted strain. The deletion of *PDC1* significantly decreased ethanol formation, although the remaining PDC activity indicated the presence of other *PDC* genes [60].

The disruption of the *CuPDC1* gene in *C. utilis* demonstrated the absence of severe growth defects. The expression of *LDH* under the native *PDC1* promoter in this strain resulted in an L-LA production of up to 103.3 g/L in 33 h on a glucose medium [62].

The introduction of bovine *LDH* into the *K. phaffii* strain with a deleted *PDC* gene resulted in an L-LA yield of 0.65 g/g [68].

*Pichia kudriavzevii* (*Issatchenkia orientalis*), with inhibited ethanol production due to the replacement of the *PDC1* gene with bacterial *LDH*, produced up to 112 g/L of D-LA in YPD medium with 15% glucose buffered with calcium hydroxide [63]. In the study by Zhang et al. [64], the deletion of *PDC* was one of the modifications made to L-LA-producing *P. kudriavzevii* that allowed for obtaining a strain producing 74.57 g/L of L-LA in a bioreactor without medium neutralization. Another engineered strain of *P. kudriavzevii* with deleted *PDC* and the heterologous expression of bacterial D-LDH was reported to produce up to 81 g/L of D-LA on sorghum hydrolysate buffered with CaCO_3_ during shake-flask fermentation [65].

*K. marxianus* possesses one *KmPDC1* gene, the disruption of which is sufficient to prevent ethanol biosynthesis. The introduction of LDH into the *K. marxianus* strains deficient in PDC activity partially restores the reduced growth rate demonstrated by such strains. Through the expression of bacterial *LDHs* in *K. marxianus* with an inactive *PDC1* gene, the production of 46.3 g/L of L-LA and 40 g/L of D-LA was achieved [54].

In *Zygosaccharomyces bailii*, the presence of at least three *PDC* genes (*ZbPDC1*, *ZbPDC2*, *ZbPDC3*) was described. Integrating fungal *LDH* into the *ZbPDC1* locus allowed for obtaining two L-LA-producing strains with reduced or eliminated ethanol production. The later-engineered strain, named ZBL, also demonstrated slowed glucose consumption and cell growth rate. The ZBL strain was able to restore ethanol production after 50 h of fermentation; however, the supplementation of the culture medium with ethanol eradicated this effect. During flask fermentation, the ZBL strain produced 35 g/L of L-LA with a yield of 0.35 g/g glucose [81].

#### 5.2.2. Alcohol Dehydrogenase

Alcohol dehydrogenase (ADH; E.C. 1.1.1.1) is another enzyme directly involved in ethanol biosynthesis, which converts acetaldehyde to ethanol regenerating NAD^+^ from NADH. Among the several ADH isozymes described in *S. cerevisiae*, ADH1 corresponds to most of the ADH activity [89]. The deletion of only *ADH1* in *S. cerevisiae* is unable to completely inhibit ethanol formation from glucose due to the upregulation of other ADH isozyme genes. This also leads to increased NADH oxidation through elevated glycerol formation [72,77,99]. In the same way, the deletion of each *ADH* gene in *K. marxianus* does not affect ethanol production and cell growth [54]. To eliminate ethanol production, the deletion of all the *ADH* isozyme genes is required.

The simultaneous deletion of *PDC1* and *ADH1* genes in *S. cerevisiae* expressing bovine *LDH* increased L-LA yield from 0.45 g/g to 0.69 g/g compared to the strain with only *PDC1* deletion [75]. The obtained strain produced 74.1 g/L of L-LA and was able to sustain growth on a minimal medium containing glucose.

#### 5.2.3. Pyruvate Dehydrogenase

The pyruvate dehydrogenase complex (PDH, EC 1.2.4.1) facilitates pyruvate conversion into acetyl-CoA. In case of a disruption of PDH activity, the acetaldehyde formed from pyruvate by PDC can still be channeled towards the TCA cycle via an alternative and less energetically efficient PDH bypass [59,100].

Bianchi et al. [59] demonstrated the effect of PDH activity disruption on L-LA production in engineered *K. lactis*. It allowed for reaching an L-LA titer of 60 g/L and a yield of 0.85 g/g in the *K. lactic* strain lacking both PDC and PDH activity, compared to the strain with only PDC disruption and an L-LA yield of 0.58 g/g. The strain with the deletion of both the *KlPDC1* and *KlPDA1* genes encoding for the E1α subunit of the PDH complex, however, could not use just C6 sugars or C3 compounds for growth on a minimal medium, thus the fermentation had to be performed on a medium with both glucose and ethanol.

### 5.3. Prevention of Glycerol Formation

Inhibited ethanol production results in an excess of NADH, hence elevated glycerol biosynthesis occurs to restore the redox balance [86]. Glycerol 3-phosphate dehydrogenase (GPD; EC 1.1.1.8) is an NADH-dependent enzyme that reduces dihydroxyacetone phosphate to glycerol-3-phosphate, which is then dephosphorylated to glycerol. *GPD* deletion aims to decrease the amount of carbon converted into glycerol.

Deleting *GPD1* and *GPD2* genes encoding GPD in D-LA-producing *S. cerevisiae* successfully eliminated glycerol production and increased D-LA production from 8 g/L to 12 g/L [77]. The same strategy for eliminating by-product formation was successfully implemented for engineering LA-producing strains including *K. marxianus* and *P. kudriavzevii* (Table 1) [65,67,76].

### 5.4. Control of Cytosolic Redox Balance

The regulation of cytosolic redox balance is one of the strategies applied to improve target metabolite production in engineered strains. It is also crucial for optimizing LA biosynthesis, considering an appropriate NADH supply is needed for the conversion of pyruvate to LA (Figure 2). By enhancing NADH regeneration pathways or modulating NADH-consuming reactions, redox balance can be shifted to favor lactate production.

NADH dehydrogenase (EC 1.6.5.9) is located in the inner mitochondrial membrane and is responsible for the oxidation of cytosolic NADH. Together with GPD, it is one of the key components for maintaining cytosolic redox balance during growth under aerobic conditions. The *NDE1* and *NDE2* genes of external mitochondrial membrane-bound NADH dehydrogenase were deleted in the L-LA-producing *S. cerevisiae* strain to shift cytosolic NADH content [76]. Under oxygen-limited flask cultivation conditions, the deletion of the *NDE1* gene increased cytosolic NADH concentration, along with ethanol and glycerol production and cellular respiration; however, L-LA production was inhibited. The further introduction of an additional copy of the *LDH* gene and the deletion of the *NDE2* gene improved L-LA production by the engineered strain.

The reversed strategy of decreasing cytosolic NADH content in engineered yeast strains was explored by Li et al. [78]. To investigate the effect of reducing the cytosolic NADH/NAD+ ratio on L-LA production, the genes of heterogeneous and endogenous NADH- or NADP-dependent enzymes (including bacterial 3-phosphate glyceraldehyde dehydrogenase (gapN), bacterial NADH oxidase (Nox), mitochondrial and cytoplasmic transporter MDH3, and glutamate synthase GLT1) were expressed in L-LA-producing *S. cerevisiae* strains. Among the studied approaches, the overexpression of *GLT1* was the most successful in reducing NADH/NAD+ ratio, and resulted in an L-LA titer increase to 37.94 g/L with a yield of 0.66 g/g on glucose, accompanied by a decrease in ethanol and glycerol formation.

### 5.5. Inhibition of LA Consumption

To avoid LA utilization by the cells, the enzymes catalyzing LA oxidation have to be eliminated. Cytochrome b2 or cytochrome c oxidoreductase (flavocytochrome b2; EC 1.1.2.3) is an enzyme located in the mitochondrial intermembrane space that facilitates the reversible conversion of L-LA into pyruvate, with cytochrome c as a co-substrate instead of NADH in aerobic conditions. The deletion of the cytochrome c oxidoreductase-coding gene (*CYB2*) in *S. cerevisiae* prevents L-LA consumption and growth on L-LA as a sole carbon source [76,90].

L-LA-producing *K. phaffii* with *CYB2* knockout demonstrated poor growth on L-LA and an almost threefold reduced specific L-LA consumption rate [69]. This strain was also characterized by enhanced growth and L-LA production. A similar effect was reported in the LA-producing *K. marxianus* [54]. The strain lacking the *CYB2* gene demonstrated an increase in L-LA titer by 12%.

D-lactate dehydrogenase (DLDH; EC 1.1.2.4) is another mitochondrial enzyme present in yeast that can convert D-LA to pyruvate. In *S. cerevisiae*, it is encoded by the *DLD1* gene [91]. The disruption of the *DLD1* gene in D-LA-producing *S. cerevisiae* caused a stronger inhibition of D-LA consumption than disruption of the *JEN1* gene [77]. In D-LA-producing *K. marxianus* with the deletion of the *DLD1* gene, the D-LA titer was increased by 26% [54].

### 5.6. Lactic Acid Transporters

One of the most limiting factors of LA production is the inhibiting effect of LA accumulation, which is mostly based on the disruption of functions of cellular membranes [85]. To resolve issues related to LA accumulation, tolerance to this organic acid must be increased. Higher LA tolerance is beneficial for LA-producing strains, since it provides better performance under acidic stress conditions. Tolerance to acidic stress is complex and associated with many different mechanisms, including LA transport.

In *S. cerevisiae*, two LA transporters have been described, Jen1 and Ady2. Jen1 facilitates lactate-proton symport, and transports monocarboxylic acids (pyruvate, acetate, and propionate), selenite, and 3-bromopyruvate [85]. *S. cerevisiae* and *K. phaffii*, with overexpressed *JEN1*, demonstrate an increase in ingoing LA transport, while the deletion of *JEN1* leads to impaired growth on LA [101,102]. The role of Jen1 in LA export is supported by the observation of moderately elevated L-LA production on glucose in engineered *S. cerevisiae* with the overexpression of *JEN1* [73,79,103]. In contrast, *JEN1* overexpression does not have the same impact on L-LA production from xylose [104]. The disruption of *JEN1* in *S. cerevisiae* results in a partial reduction in LA consumption after glucose depletion in the culture medium, but has no significant effect on LA production [73,77,103].

Ady2 is another proton–anion symporter responsible for transporting carboxylic acids (lactate, acetate, pyruvate, propionate, and formate) and ammonia in *S. cerevisiae* [85]. It presents a target for modifications to improve LA production considering that the role of Ady2 in LA transport is similar to that of Jen1; however, its affinity for LA is 7-fold lower [103].

It is suggested that more mechanisms are involved in LA transport in yeast given that the double deletion of *JEN1* and *ADY2* is unable to eliminate LA export, but they still have yet to be identified [103,104].

## 6. Adaptive Laboratory Evolution as a Selection Tool for Improved LA-Producing Strains

Adaptive laboratory evolution (ALE) is a common method for obtaining microbial strains with the desired phenotype, which relies on the accumulation of spontaneous mutations under laboratory conditions tailored for positive selection. The results of ALE are unpredictable, and depend on the applied selective pressure and the duration of the experiment. In the case of engineering LA producers, ALE allows for obtaining strains with higher growth rates on selected substrates and increased LA tolerance. Additionally, screening for mutations in ALE-derived strains helps identify potential targets for metabolic engineering [105].

The D-LA-producing *S. cerevisiae* strain demonstrated enhanced glucose consumption and D-LA production compared to the parental strain after serial cultivation on medium with a gradual increase in D-LA from 1.3 to 3.95% [77].

In the study by Zhu et al. [79], an engineered *S. cerevisiae* strain was subjected to ALE during 12 serial subcultures, with a gradual increase in L-LA concentration from 10 g/L to 60 g/L. This led to a 17.5% increase in L-LA titer, a 42.1% increase in cell growth, and a 50.6% increase in glucose consumption compared to a parental strain. Furthermore, the increase in ethanol production by 27.2% was described.

The adaptive laboratory evolution of the L-LA-producing *O. polymorpha* strain allowed an increase in the growth rate on methanol by 55% compared to the unevolved strain, and allowed the testers to achieve an L-LA titer of 3.8 g/L during a methanol-fed batch cultivation in shake flasks, using urea as a nitrogen source [70].

While ALE has been proven to be a powerful tool for improving strain tolerance and productivity, it is inherently limited by the randomness of mutations, time requirements, and the challenge of translating lab-scale improvements to industrial conditions [105]. The selection conditions must be carefully optimized to avoid unintended trade-offs in desirable traits. Despite the precision offered by tools like CRISPR-Cas9, gene editing faces challenges including off-target effects, metabolic pathway complexities, and the regulatory hurdles associated with GMO applications. Ensuring the stability of genetic modifications over long fermentation periods and under industrial conditions remains another critical challenge. The integration of ALE and gene editing offers a promising avenue for strain optimization. ALE can identify novel genetic targets, which can then be fine-tuned using CRISPR or similar tools, enhancing the precision and efficiency of strain improvement [106].

For example, an L-LA-producing *S. cerevisiae* strain isolated after several rounds of ALE demonstrated better growth on a medium, with 8% L-LA. The genome sequencing of the studied strain helped identify potential targets for the metabolic engineering of more tolerant LA producers. Although neither the introduction nor deletion of the investigated genes in the parental strain affected L-LA tolerance, their deletion in the evolved strain resulted in lower L-LA tolerance [107]. Another study of an evolved D-LA-producing *S. cerevisiae* strain using reverse engineering demonstrated that combined *ERF2* deletion and *SUR1^I245S^* mutation improved glucose uptake, D-LA production, and D-LA tolerance [77]. These findings support the observation that LA tolerance is a complex quality arising from multiple factors that can be identified using a combination of random mutagenesis and targeted engineering.

## 7. Challenges and Future Perspectives

Currently, the application of yeast for LA production faces several challenges. Due to the high content of pure sugars and the processing efficiency of first-generation feedstock, yeast is the most common substrate used for large-scale LA production. It raises concerns from an environmental, economic, and social point of view, considering the competition for pure sugars and edible crops between food production and industrial purposes. Challenges associated with first-generation feedstocks include soil degradation, water scarcity, and pollution from the excessive use of water, fertilizers, and pesticides [108]. Recently, the need to switch to renewable, cheap, and abundant substrates has attracted a lot of attention to alternative options, including lignocellulose, starchy materials, agro-industrial and food waste, glycerol, methanol, etc. They usually contain enough carbohydrates and other nutrients to sustain LA fermentation. The perspectives on LA production by yeast using alternative substrates were demonstrated, but further optimization is required for effective commercial use [4,108].

Lignocellulose is a highly abundant plant-derived substrate that contains pentoses, notably xylose and arabinose, along with hexoses, mainly glucose [109]. The spectrum of lignocellulosic waste that can be used for LA production is vast. Studies have demonstrated the use of metabolically engineered yeasts for LA production from various types of biomass waste, such as *S. cerevisiae* utilizing spent coffee grounds and sugarcane bagasse [110]. Despite the environmental advantages, the use of lignocellulosic plant biomass for LA production is complicated due to certain challenges. The first one emerges from the inability of most yeasts to simultaneously metabolize a wide range of sugars, leading to low LA yield. The solution to this problem calls for the application of different yeast species and the metabolic engineering of respective pathways to improve the efficiency of substrate utilization. The efficient utilization of xylose to L-LA has already been demonstrated in several yeast strains, including *P. stipitis* [111], *S. cerevisiae* [112,113,114,115], *C. utilis* [116] and *C. sonorensis* [117]. Kong et al. engineered a *K. marxianus* strain able to co-ferment xylose and glucose from corncob hydrolysate with a high yield of optically pure L-LA [67]. Lignocellulosic plant waste as well as other alternative substrates also often require physicochemical or enzymatic pretreatment, resulting in the formation of by-products such as organic acids, phenolic compounds, and aldehydes that can act as inhibitors negatively impacting cell growth, substrate utilization, and the fermentation process [118]. Addressing these challenges requires either the detoxification of lignocellulose hydrolysates through chemical or biological methods or the engineering of yeast strains with enhanced inhibitor tolerance. *K. marxianus*, for example, has been shown to metabolize lignocellulosic hydrolysates effectively while tolerating inhibitors, making it a promising candidate for sustainable LA production [54,67].

Inhibition may arise from the substrate itself, as high sugar concentrations can reduce enzymatic activity during lignocellulosic hydrolysis, lowering sugar formation rates and inhibiting cell growth [119]. Applying simultaneous saccharification and fermentation (SSF) to lignocellulosic biomass utilization, thus synchronizing the breakdown of complex sugars and fermentation, reduces feedback inhibition and increases process efficiency [120].

The products of LA fermentation could demonstrate inhibiting properties, penetrating cell membranes and inducing a decrease in intracellular pH [85]. Inhibition by LA introduces the need to use neutralizing agents, as it generates more waste at the final stages of industrial LA production [114]. This limitation can be avoided by exploring potential microbial LA producers with naturally high tolerance to low-pH environments, and improving the tolerance of existing LA-producing strains by targeted genetic modification or random mutagenesis [63,79]. Another challenge arises from the processes of the recovery and purification of LA that involve the application of expensive reagents and equipment generating a lot of waste. The solution to these obstacles combines the optimization of fermentation modes, the removal of LA from the fermentation medium, and the detoxification of hydrolysates using various chemical and biological methods. All the mentioned issues apply to LA production on both first-generation and second-generation substrates, and contribute to high manufacturing costs, making LA production unsustainable. This drives the active exploration of biotechnological solutions that have the potential to make yeasts leading LA producers for industrial purposes, especially in the growing market of PLA materials [16,121].

In summary, the global LA market is growing steadily due to its increasing and wide-ranging applications, starting from the food market and reaching to the medical, pharmaceutical, cosmetic, chemical, textile, and polymer markets. This brings about a constant need to develop new methods. Most studies on LA production are based on LAB; however, yeasts should be considered a future avenue of efficient and sustainable LA production. Whether metabolically engineered natural producers, like *L. thermotolerance*, or yeasts with heterologous *LDH* genes are the most effective should be further investigated.

## Figures and Tables

**Table 1:** Lactic acid production by engineered yeast strains.

Yeast	LA Isomer	*LDH* Source	Additional Modifications	LA Titer, g/L	LA Yield, g/g	Carbon Source	Reference
*Candida boidinii*	L	*Bos taurus*	*∆pdc1*	86	1	Glucose	[60]
*Candida sonorensis*	L	*Lactobacillus helveticus*	*∆pdc1::LhLDH*, *∆pdc2*	92	0.94	Glucose	[61]
*Candida utilis*	L	*B. taurus*	*∆pdc1::LDH2*	103.3	0.95	Glucose	[62]
*Pichia kudriavzevii (Issatchenkia orientalis)*	D	*Lactobacillus plantarum*	*∆pdc1*	112	ND	Glucose	[63]
*P. kudriavzevii*	L	*Weizmannia coagulans*, *B. taurus*	*∆pdc1*, *∆dld*	74.57	0.93	Glucose	[64]
*P. kudriavzevii*	D	*Leuconostoc mesenteroides*	*∆pdc*, *∆gpd*, expression of *XR*, *XDH*, *XK*	80.8	ND	Sorghum hydrolysate	[65]
*Kluyveromyces lactis*	L	*B. taurus*	*∆pdcA*	109	ND	Glucose	[66]
*K. lactis*	L	*B. taurus*	*∆pdc1*, *∆pda1*	60	0.85	Glucose	[59]
*Kluyveromyces marxianus*	D	*L. plantarum*	*∆pdc1*, *∆cyb2*	122	0.95	Jerusalem artichoke powder (JAP)	[54]
	L	*L. plantarum*	*∆pdc1*, *∆dld1*	130	0.98		
*K. marxianus*	L	*Plasmodium falciparum*, *Bacillus subtilis*	*∆dld1*, *∆gpd*, overexpression of *KmPFK*, *ScJEN1*	125.93	0.63	JAP	[67]
*Komagataella phaffii*	L	*B. taurus*	*∆pdc1*	30	0.65	Glycerol	[68]
*K. phaffii*	L	*L. plantarum*	*∆cyb2*, CO_2_ fixation pathway	0.2	ND	CO_2_	[69]
*Ogataea polymorpha*	L	*L. helveticus*	ALE	3.8	0.08	Methanol	[70]
*Saccharomyces cerevisiae*	L	*Lactobacillus casei*		12	ND	Glucose	[71]
*S. cerevisiae*	L	*Rhizopus oryzae*	*∆adh1*	17	ND	Glucose	[72]
*S. cerevisiae*	L	*L. plantarum*	*JEN1* overexpression	8	0.39	Glucose	[73]
*S. cerevisiae*	L	*B. taurus*	*∆pdc1*, *∆pdc5*	82.3	ND	Glucose	[74]
*S. cerevisiae*	L	*B. taurus*	*∆pdc1*, *∆adh1*	74.1	0.69	Glucose	[75]
*S. cerevisiae*	L	*Pediococcus sinensis*	*∆pdc1::ldh*, *∆cyb2::ldh*, *∆gpd1::ldh*, *∆trp1::ldh*, *∆nde1*, *∆nde2::ldh*	117	0.58	Glucose	[76]
*S. cerevisiae*	D	*L. mesenteroides*	*∆dld1*, *∆jen1*, *∆adh1*, *Δgpd1*, *∆gpd2*, *∆pdc1::Lm.ldhA*, expression of *HAA1* ALE	112	0.8	Glucose	[77]
*S. cerevisiae*	L	*B. taurus*	*Δpdc1*, *Δadh1*, overexpression of *ALD6*, *ACS1*, *GLT1*	37.94	0.66	Glucose	[78]
*S. cerevisiae*	L	*B. taurus*	*∆pdc1*, *∆adh1*, expression of *eutE*, *JEN1*, ALE	121.5	0.81	Glucose	[79]
*S*. *cerevisiae*	L	*Lactococcus* *lactis*	*Δcyb2::LacLDH*, *Δpdc1::LacLDH*, *Δpdc6::LacLDH*, *Δadh1::LacLDH*, *Δgpd1::LacLDH*, *Δgpd2::LacLDH*, *Δjen1::LacLDH*, expression of *ADY2*, *Δnde1*, *Δnde2::pfkA*	192.3	0.78	Glucose	[80]
*Zygosaccharomyces bailii*	L	*R. oryzae*	*∆pdc1::LDH*	35	0.35	Glucose	[81]

ND—not determinated.
